# Testing the 4Rs and 2Ss Multiple Family Group intervention: study protocol for a randomized controlled trial

**DOI:** 10.1186/s13063-017-2331-7

**Published:** 2017-12-04

**Authors:** Mary Acri, Emily Hamovitch, Maria Mini, Elene Garay, Claire Connolly, Mary McKay

**Affiliations:** 1 McSilver Institute for Poverty Policy and Research, New York University Silver School of Social Work, 41 East 11th Street, 7th floor, New York, NY 10003 USA; 2 George Warren Brown School of Social Work, Washington University, 1 Brookings Drive, St. Louis, MO 63130 USA

**Keywords:** Implementation and sustainability, Child mental health, Oppositional defiant disorder, Family functioning

## Abstract

**Background:**

Oppositional defiant disorder (ODD) is a major mental health concern and highly prevalent among children living in poverty-impacted communities. Despite that treatments for ODD are among the most effective, few children living in poverty receive these services due to substantial barriers to access, as well as difficulties in the uptake and sustained adoption of evidence-based practices (EBPs) in community settings. The purpose of this study is to examine implementation processes that impact uptake of an evidence-based practice for childhood ODD, and the impact of a Clinic Implementation Team (CIT)-driven structured adaptation to enhance its fit within the public mental health clinic setting.

**Methods/design:**

This study, a Hybrid Type II effectiveness-implementation research trial, blends clinical effectiveness and implementation research methods to examine the impact of the 4Rs and 2Ss Multiple Family Group (MFG) intervention, family level mediators of child outcomes, clinic/provider-level mediators of implementation, and the impact of CITs on uptake and long-term utilization of this model. All New York City public outpatient mental health clinics have been invited to participate. A sampling procedure that included randomization at the agency level and a sub-study to examine the impact of clinic choice upon outcomes yielded a distribution of clinics across three study conditions. Quantitative data measuring child outcomes, organizational factors and implementation fidelity will be collected from caregivers and providers at baseline, 8, and 16 weeks from baseline, and 6 months from treatment completion. The expected participation is 134 clinics, 268 providers, and 2688 caregiver/child dyads. We will use mediation analysis with a multi-level Structural Equation Modeling (SEM) (MSEM including family level variables, provider variables, and clinic variables), as well as mediation tests to examine study hypotheses.

**Discussion:**

The aim of the study is to generate knowledge about effectiveness and mediating factors in the treatment of ODDs in children in the context of family functioning, and to propose an innovative approach to the adaptation and implementation of new treatment interventions within clinic settings. The proposed CIT adaptation and implementation model has the potential to enhance implementation and sustainability, and ultimately increase the extent to which effective interventions are available and can impact children and families in need of services for serious behavior problems.

**Trial registration:**

ClinicalTrials.gov, ID: NCT02715414. Registered on 3 March 2016.

**Electronic supplementary material:**

The online version of this article (doi:10.1186/s13063-017-2331-7) contains supplementary material, which is available to authorized users.

## Background

Oppositional defiant disorder (ODD) is a chronic and impairing mental health disorder that afflicts between 1 and 21% of the population, and primarily elementary school-age children, with the median age of onset being 11.6 years [1]. Along with conduct disorder and attention deficit hyperactivity disorder, ODD is part of the disruptive behavior disorder (DBD) diagnostic group, which is characterized by severe behavioral problems including impulsivity, aggression, violence, delinquency, and criminal acts [1].

Family poverty is one of the greatest risk factors for the onset and perpetuation of DBDs [2–4]: studies indicate that the accumulation of risk factors associated with poverty, including increased exposure to violence and traumatic events, devastating impact of substance abuse, criminal activity, and chronic disease, as well as family strain have an additive effect, in that the more risk factors a child is exposed to increases their likelihood of exhibiting serious behavioral challenges [1, 5]. Accordingly, children living in poverty experience DBDs at rates of up to four times higher than children reared in families with more financial resources [6].

Treatments for DBDs are among the most well-studied and efficacious [7], yet as many as 80% of children in poverty-impacted communities do not receive services. And for those who do manage to initially engage in behavioral health care, premature attrition from services approaches 50% [1, 8]. Further, the uptake of evidence-based practices (EBPs) in community-based settings is slow and beset with challenges. An oft-cited statistic suggests that it can take as long as two decades before treatments that are studied in research trials become integrated as part of standard practice in real-world settings [9] if at all. Thus, even though children who live in poor communities are at the highest risk for DBDs, they are the least likely to receive effective mental health services.

### The 4Rs and 2Ss Multiple Family Group (MFG) intervention

The 4Rs and 2Ss Multiple Family Group (4Rs 2Ss MFG) for Strengthening Families Program is an evidence-based, manualized, family based treatment for families of children with disruptive behaviors [10–15]. Recently listed on the Substance Abuse and Mental Health Services Administration (SAMHS) National Registry of Evidence-based Programs and Practices [16], this model is associated with high attendance rates, improvement in behavior problems, caregiver depression and stress among predominantly low-income, minority families [14].

### Conceptual model

The 4Rs and 2Ss MFG intervention draws from group therapy techniques, systemic family therapy, and behavioral parent training [12]. It was developed using a common elements approach [10], which entails identifying techniques and procedures that are common to already existing evidence-based protocols for specific problem areas [17]. As such, the 4Rs and 2Ss MFG integrates family processes and parenting skills linked to conduct problems from the empirical academic literature [10, 14]. The targeted skills and processes are referred to in the curriculum as the 4Rs (Rules, Responsibility, Relationships, and Respectful Communication) and 2Ss (Stress and Social support) [14]. Additionally, families of youth with DBDs and mental health providers assisted in the model’s development specific to enhancing cultural and contextual relevance of the intervention content, as well as engagement [10].

### Implementation processes

Although positive outcomes from the 4Rs and 2Ss MFG were found in prior studies, significant implementation challenges emerged [18]. Ongoing leadership support varied, and provider motivation waned in the presence of high levels of family engagement and intensity of family need. These findings suggested that the implementation of the 4Rs and 2Ss MFG required attention to multi-level implementation processes to modify service delivery practices on a sustainable level.

There has been a growing interest within the mental health field in implementation processes that facilitate the uptake and adoption of evidence-based practices in community-based settings [19, 20]. The adoption of EBPs is predicated upon both inner contextual factors pertaining to the organization, including the agency’s climate, leadership support, and provider perceptions about EBPs [21–26], and the larger external context in which the organization is embedded [23], including the political and legislative landscape that may mandate or enforce specific practices, and reimbursement mechanisms [21].

Hybrid effectiveness-implementation research designs were developed to focus both on an intervention’s effectiveness and implementation processes, either sequentially or simultaneously, which is proposed to accelerate the process to integrating EBPs in real-world practice settings [27]. As noted by Curran [27] there are three types of hybrid designs; examination of the intervention’s effects while observing implementation processes, examination of implementation processes while observing effectiveness, and examining both effectiveness and implementation simultaneously. This study will employ the latter, a Hybrid Type II design, which tests both clinical and implementation areas simultaneously [27].

### Purpose of this study

**Fig. 1:**
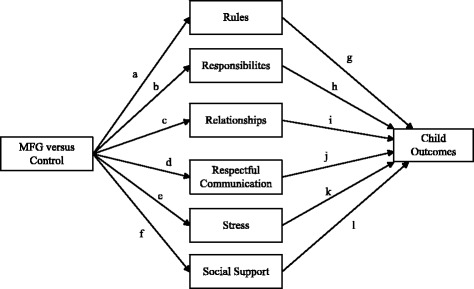
Analysis of mediation

This study reflects a combination of clinical effectiveness and implementation research methods, with aims focused on replicating child outcomes associated with the 4Rs and 2Ss MFG, and gathering information about family and clinic/provider-level moderators of implementation. A secondary objective is to test an innovative method of adaptation of the 4Rs 2Ss MFG through the formation of change teams that are hypothesized to enhance outcomes and inner contextual factors. Within the academic literature on implementation processes, there is a growing emphasis upon staff serving as champions or change agents to implement EBPs and address potential barriers to adoption [25, 28, 29, 35]. In this study, providers and leadership will form a Clinic Implementation Team (CIT) and will be guided through a standardized process by which they adapt the intervention’s content, format, and structure. As shown in Fig. 1, a main premise of this study is that adaptation of 4Rs and 2Ss MFG by the CIT will impact clinic-level mediators of readiness, leadership support, and climate (path m) which, in turn, is proposed to affect provider-level variables (path n). These differential effects then carry through to the core family variables (path o) and, in turn, child outcomes (path p).

## Methods/design

### Study aims

In 2014, the National Institute of Mental Health (NIMH)-funded “Family Groups for Urban Youth with Disruptive Behavior.” The grant began with a planning stage in 2015 and will be completed by 2019. The four aims of the study are to examine:

1. The short-term and longitudinal impact of the 4Rs and 2Ss MFG model on child ODD and functioning
2. Family level mediators, such as parenting and family processes, that impact child outcomes
3. Clinic- and provider-level factors (organizational culture and climate, leadership support, provider attitudes towards EBPs) upon implementation of the 4Rs and 2Ss MFG implementation, and
4. The impact of Clinic Implementation Teams on clinic- and provider-level moderators of 4Rs and 2Ss MFG implementation and integration

### Participants

All NYS Office of Mental Health (OMH) child-serving public outpatient mental health clinics across the five boroughs of New York City (NYC) are eligible to participate. To secure the sample of clinics, the study team obtained a master list of 168 clinics from OMH. Based on prior statewide studies conducted by the investigative team, it was estimated that approximately 80% of the clinics would participate; thus, we estimated that approximately 134 clinics will enroll in the study.

At each clinic, we anticipate enrolling 20 caregiver/child dyads (a minimum of *n* = 896 per arm, or 2688 total), and two providers per arm, yielding a total of 268 providers. Researchers will consecutively consent families to participate in the study until a sample of 20 caregiver/child dyads per clinic is achieved. Eligible caregivers are 18 years of age or older, English or Spanish speaking, and the primary caregiver of a 7- to 11-year-old child with ODD. Eligible providers are staff members who work in a participating clinic in a clinical capacity, and are English speaking.

### Sampling procedure

A multi-stage sampling procedure was undertaken, with randomization occurring at the agency level in order to prevent an unbalanced representation of clinics across the treatment arms and to conduct a sub-study in which we will examine the impact of clinic choice upon outcomes. From a master list of OMH-licensed agencies and corresponding clinics within the five boroughs of NYC that was received from The Office of Mental Health in 2016, the investigative team created three lists: (1) single-site (agencies that had only one outpatient mental health clinic, (2) multi-site agencies with between two to four clinics and, (3) mega-agencies that had five or more clinics. Each list was organized alphabetically.

A separate randomization process was employed for each list. Clinics within single-site agencies were stratified by borough, and a random number generator was employed to randomly assign clinics to one of three conditions: (1) condition 0, which was services as usual (SAU), (2) condition 1, the 4Rs and 2Ss MFG, and (3) condition 2, the 4Rs and 2Ss MFG + CIT (all three conditions are described more thoroughly on page 8). Once the initial number was generated, subsequent clinics were assigned the next number in the sequence (e.g., if the first clinic received condition 0, the next clinic would be enrolled in condition 1, and so forth). Of note, the research staff member was unaware what numbers corresponded with which condition (e.g., that a clinic in condition 0 would be randomized to SAU). This procedure was undertaken in order to minimize allocation bias.

Multi-site agencies with between two and four clinics were also organized alphabetically within borough, and a random number generator was used to assign the initial clinic to a particular condition. Moreover, additional clinics that were part of the agency in which the initial clinic was randomized received the same condition. For example, if one clinic was assigned condition 0, the remaining clinics, regardless of which borough they were located in, were also assigned to condition 0.

Finally, agencies with five or more clinics were given the choice to select the study condition in which they would participate with an even distribution of these mega agencies occurring across the three study conditions.

### Description of the conditions

The study consists of three arms: two active treatment and one comparison condition:

1. The 4Rs and 2Ss Multiple Family Group intervention
  The 4Rs and 2Ss MFG is a 16-week group for urban children of between 7 and 11 years of age with ODD and their caregiver(s). In this model, six to eight families meet in weekly sessions focused on addressing family and parenting factors associated with behavior problems [30–33]. Session content also addresses lack of social support and high stress, which are two factors known to hinder treatment attendance [34–36]. Families are provided with free childcare, transportation expenses, and a meal to address common logistical barriers to attendance [8]. Moreover, perceptual barriers, such as stigma and fears of being blamed for their child’s difficulties, are targeted through group processes which normalize family difficulties, validate their expertise in solving problems, and promote mutual aid [37]. Providers who participate in this condition will attend a 5-h training to learn the 4Rs and 2Ss MFG model; thereafter, they will participate in bimonthly supervision over the course of implementation
2. The 4Rs and 2Ss MFG + CIT
  In addition to implementing the model and receiving supervision, a CIT consisting of providers, a clinic director and supervisor will participate in ongoing meetings with the research team’s adaptation specialist for a guided analysis of the structure, content and processes of the treatment model, including those that are amenable to modification and those that must remain unchanged in order to maintain fidelity
3. Standard care
  Standard care includes the services that the clinic normally provides to children with behavioral problems, including outpatient mental health therapy and pharmacology

### Fidelity and supervision

Group facilitators will provide supervision by phone on a bimonthly basis, and three face-to-face supervision meetings after each fidelity check. Supervision will be conducted by clinical staff who have extensive knowledge of the 4Rs and 2Ss model, and who have supervised clinicians in other initiatives using this model. Additionally, two independent raters will conduct fidelity checks for a minimum of three sessions to ensure adherence to the treatment model. As fidelity checks will be conducted by two personnel, inter-rater reliability will be computed. Providers falling below 80% in fidelity will be given additional supports, such as additional supervisory sessions, to improve adherence.

### Clinic involvement

Of the approximately 134 clinics that will be invited to participate; 37 clinics have been enrolled in the study thus far. Data collection has begun at 14 clinics in the SAU clinic, eight clinics in condition 1 and seven clinics in condition 2. Others have agreed to participate and data collection will begin shortly.

### Measures

Data collection will occur at baseline, 8, and 16 weeks from baseline, and at 6-month follow-up. Demographic characteristics about familial factors (e.g., age, race/ethnicity, family income) will be collected via a questionnaire used in prior studies. A separate demographic questionnaire will be administered to providers to gather information including their age, race/ethnicity, education, and credentials.

### Child factors

#### Child oppositional defiant disorder

will be measured by the Disruptive Behavior Disorders Rating Scale Oppositional Defiant Subscale [38]. Completed by the child’s caregiver, this subscale consists of eight items that are ranked using a 4-point Likert scale ranging from “not at all” (0) to “very much” (3). Total scores range from 0 to 24, and a total of four or more items endorsed as “pretty much” or “very much” are needed to meet criteria for ODD. Two studies that used this scale across several outpatient mental health clinics within NYC found good internal consistency with a Cronbach’s *α* at baseline of .70 and .89, respectively [39].

Child ODD will also be measured by the Iowa Conners Rating Scale-Oppositional/Defiant Subscale (IOWA CRS OD) [40], a widely used brief measure of oppositional defiant behavior in children. Completed by the child’s caregiver, the IOWA CRS OD includes items ranked using a 4-point Likert ranging from “not at all” (0) to “very much” (3). Total scores range from 9 to 30, with higher scores indicating greeter severity of symptoms. A previous randomized effectiveness study of the MFG intervention found that this scale had good internal consistency with a Cronbach’s *α* at baseline of .86 [39].

#### Functioning

Child functioning will be measured via the Impairment Rating Scale (IRS), [41] a six-item instrument that asks caregivers to rate the severity of their child’s problems and need for treatment across functional domains, including their relationship with peers, parent(s), and sibling(s); academic progress, and family functioning. Caregivers place an “X” on a 7-point scale to signify their child’s functioning along a continuum of impairment that ranges from 0 (no need for treatment) to 6 (extreme need for treatment). Scores of three or greater indicates clinical impairment [42]. The IRS has shown evidence for concurrent, discriminant, and convergent validity, and acceptable levels of temporal stability [43].

### Caregiver factors

#### Depression

Caregiver depression will be measured using the short form of the Center for Epidemiologic Studies Depression Scale (CESD) [44-46], a free, publically available screening tool consisting of seven items that assesses the frequency of depressive symptoms within the past week. Items are anchored on a 4-point Likert scale ranging from 0 (rarely/none of the time) to 3 (most or all of the time). CESD scores range from 0 to 21, with a score of 8 and above considered clinically significant depressive symptoms [45]. The CESD short form evidences high internal consistency, with a Cronbach *α* of above 0.8 [45]

#### Stress

The Parenting Stress Index (PSI) short form will be administered to measure stress [47]. This 36-item scale uses a 5-point Likert scale, ranging from “strongly disagree (1)” to “strongly agree (5).” Total scores range from 36 to 180, with higher scores indicating increased levels of parent stress [48]. In a prior study of the 4Rs and 2Ss MFG, Cronbach’s *α* values reported as baseline, mid test, post test, and 6-month follow-up, were 0.91, 0.92, 0.94, and 0.94, respectively [39].

### Family factors

#### Parenting quality

The Alabama Parenting Questionnaire (APQ-9) short form will measure parenting factors, and specifically positive parenting, inconsistent discipline, and poor supervision [49]. This scale consists of nine items that are measured in terms of frequency, ranging from never (1), to always (5). Items are summed to determine positive parenting, inconsistent discipline, and poor supervision. The APQ-9 short form was shown to be reliable and valid in two prior studies, and has a Cronbach’s *α* ranging from 0.59 to 0.84 in the first study and 0.80 to 0.92 in the second study [49].

#### Uptake of the 4Rs and 2Ss MFG

Two subscales of the McMaster Family Assessment Device (FAD) [50] will measure the extent to which the 4Rs are used within the family. The two subscales, focusing on roles and communication, use a 4-point Likert scale ranging from strongly disagree (1) to strongly agree (4). Higher numbers indicate more problematic functioning. The FAD was shown to have strong validity in multiple studies, and a test of this scale using a sample of 503 individuals found acceptable reliability of the subscales, with a Cronbach’s *α* of 0.72 (Roles) and 0.75 (Communication) [50].

#### Social support

To measure social support, the family subscale of the Multidimensional Scale of Perceived Social Support (MSPSS) [51] will be administered. This four-item subscale, uses a 7-point Likert scale ranging from very strongly disagree (1) to very strongly agree (7). Scores are summed, with higher scores reflecting greater perceived support.

The MSPSS has strong factorial validity as well as good internal and test-retest reliability (Cronbach *α* = 0.85 for the Family subscale) [51].

#### Engagement in treatment

Attendance will be collected, and a total number of sessions attended will be tallied for each participant.

#### Barriers to treatment

These will be assessed using the Kazdin Barriers to Treatment (KBT) scale [52], which is comprised of three subscales: perceived relevance of treatment, relationship with the therapist, and critical events. The first two subscales consist of 14 items combined, and are rated on a 5-point scale, ranging from (1) never a problem to (5) very often a problem. The third subscale, delivered in a yes-no format, includes 14 critical events that may lead to treatment termination. Items on the 5-point scale are summed to provide the total barriers score, and items on the binary scale (1 = no, 2 = yes) are summed to reflect the absence or presence of critical events [52, 53]. Higher scores reflect greater severity regarding barriers to treatment [52].

### Implementation processes factors

#### Attitudes about evidence-based practices

The Evidence-Based Practice Attitude Scale [21] will assess provider attitudes toward evidence-based practices. The scale is comprised of four subscales: the intuitive *appeal* of EBP, the likelihood of adopting EBP in light of such *requirements*, *openness* to new practices, and the degree to which *divergence* is perceived between usual practice and research-based/academically developed interventions. Five possible responses are provided on Likert scale, from 0 (not at all) to 4 (to a very great extent). For each subscale, a mean score is calculated and the fourth (divergence) is reverse coded. A higher mean score indicates a greater degree of the construct reflected by that subscale. A study using this scale to assess service provider attitudes from 51 mental health service programs found good internal consistency reliability, with an overall Cronbach’s of 0.77 [21].

#### Organizational processes and readiness to change

This will be assessed via the Texas Christian University (TCU) Organizational Readiness for Change (TCU ORC) instrument [54]. Responses to this 66-item instrument range from strongly disagree (1) to strongly agree (5). Subscales focus on motivation for change, resources, and staff. In addition, the Training Exposure and Utilization subscale measures training exposure and utilization within an agency. It contains seven items that are assessed on a scale that ranges from never (1) to almost always (5). Mean scores for each subscale are multiplied by 10, so that final scores range from 10 to 50. Scale scores above 30 indicate some average agreement on the concept measured by the scale, and scale scores below 30 indicate some disagreement on average [55]. Reliability and validity of the ORC were examined in a study using a national sample of over 500 staff members from more than 100 programs, with the majority of subscales showing a Cronbach’s *α* of above .70 [54].

#### Beliefs about treatment

The Measure of Beliefs about Participation in Family Centered Service Delivery [56] will assess providers’ beliefs about participating in family centered services. This 28-item instrument consists of five scales that measure beliefs about family centered philosophy and principles, positive and negative outcomes, personal competencies, and barriers [56]. Responses are provided on a 7-point scale ranging from strongly disagree (1) to strongly agree (7). The total scale score is calculated as the average of all means of the scale scores. Higher scores on scales represent stronger beliefs regarding family centered services. A study examining the development and validity of this instrument using 818 respondents showed good evidence of construct validity for this measure as well as moderate to excellent reliability, with coefficient *α* values ranging from .61 to .83 [56].

#### Treatment fidelity and supervision

Fidelity monitoring tools will assess facilitator adherence to curriculum content (e.g., session topic, information conveyed, competence in guiding discussions) and clinical skills (i.e., active listening). These fidelity assessments were standardized during fidelity observations of prior 4Rs and 2Ss MFGs studies [1, 18].

#### Organizational characteristics

These include size, fiscal health and funding streams, and populations served will be analyzed from two pre-existing datasets collected by New York State Office of Mental Health (NYSOMH) about organizational composition and service delivery.

### Hypotheses and data analytic strategy

The purpose of this study is to examine outcomes associated with the 4Rs and 2Ss MFG, implementation processes that impact uptake and sustained use of the intervention over time, and the impact of an innovative change agent team upon outcomes. The following hypotheses will be tested:

- *Aim #1*: to examine the short-term and longitudinal impact of 4Rs and 2Ss MFG on child ODD and functioning
- *Hypothesis #1*: youth who participate in 4Rs and 2Ss MFG will display significantly reduced conduct difficulties and increased functioning over time compared to those involved in standard care: youth in the 4Rs and 2Ss MFG + CIT condition will evidence the greatest magnitude of change in outcomes over time
- *Aim #2*: to examine family level mediators (e.g., parenting, family process) of child outcomes
- *Hypothesis #1*: based on existing 4Rs and 2Ss MFG findings, specific family level variables (caregiver stress, the parent/child relationship and established rules) will be associated with significantly greater impact on child outcomes over time relative to social support, established responsibilities, and respectful communication
- *Aim #3*: to examine clinic- (readiness to adopt an innovation, leadership support, and climate) and provider-level moderators (engagement, preparedness, motivation) of 4Rs and 2Ss MFG implementation and integration
- *Hypothesis #1*: leadership support will evidence significantly greater impact on 4Rs and 2Ss MFG implementation/integration in comparison to general readiness and clinic climate
- *Hypothesis #2*: provider motivation will evidence significantly greater impact on 4Rs and 2Ss MFG implementation/integration relative to other provider-level variables
- *Aim #4*: to examine the impact of CITs on clinic- and provider-level moderators of 4Rs and 2Ss MFG implementation and integration
- *Hypothesis #1*: clinic directors assigned to MFG + CIT will evidence significantly higher readiness for innovation and support, and providers assigned to MFG + CIT will evidence significantly higher preparedness and motivation to implement MFG relative to MFG alone
- *Hypothesis #2*: preparedness, motivation of providers, and leadership support assigned to MFG + CIT will be maintained over time and be significantly higher relative to MFG alone
- *Hypothesis #3*: providers assigned to MFG + CIT will evidence significantly enhanced fidelity to MFG relative to MFG only
- *Hypothesis #4*: clinics assigned to MFG + CIT will have more MFGs initiated and completed (with high fidelity and attendance) relative to MFG alone

Overall, the two primary outcomes being tested in this study are changes in child behavior and child functioning. These outcomes are being tested specifically in aims 1 and 2, as measured by the Disruptive Behavior Disorders Rating Scale Oppositional Defiant Subscale [38], Iowa Conners Rating Scale-Oppositional/Defiant Subscale [40] and the Impairment Rating Scale (IRS) [41]. Aims 3 and 4 intend to measure mediators of the primary outcome, by testing clinic readiness and motivation to implement the 4Rs and 2Ss intervention initially and over time, and testing whether fidelity has been adhered to.

### Analytic strategy

**Fig. 2:**
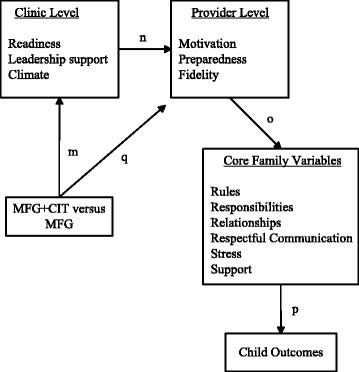
Analysis of mechanisms

The overall analytic structure uses a three-level, multi-level Structural Equation Modeling (MSEM) framework with family level variables at level 1, provider variables at level 2, and clinic variables at level 3. The analytic structure is complex, so we highlight our approach using Fig. 2 as our primary reference. The MSEM framework is useful because it ultimately allows a test of these aims within an integrated path modeling structure.

We will employ mediation analysis with two-level MSEM models as explicated by Preacher, Zhang and Zyphur [57] and extended to three-level models by Preacher [57]. We will use a combination of limited information estimation frameworks (focused on sub-portions of the full model) and full information estimation frameworks (focused on the entire three-level model) to strategically answer questions. At level 1, child outcomes at the immediate post test are modeled as a function of the family predictors (see Fig. 2) using baseline measures of outcomes and family mediators as covariates, taking into account the clustering at the higher levels of the model. The follow-up (6-month) measures of the mediators and the outcomes are included within the model in a classic SEM panel model with autoregressive effects, thereby linking the follow-up data to the immediate post-test data. This is an advantage of using MSEM over traditional multi-level modeling. Different centering strategies (e.g., grand mean versus group mean) can be explored to garner various perspectives on the data. This feature of the model provides perspectives on aim 2 and the relative importance of paths g–l in Fig. 1. To ascertain perspectives of the effects of clinic-level variables on provider variables, random intercept MSEM with the provider-level variables can be estimated as a function of the clinic variables that include dummy coded treatment variables (4Rs and 2Ss MFG + CIT versus 4Rs and 2Ss MFG) impacting the clinic mediators of readiness, leadership support, and climate, which in turn, affect the provider-level intercepts. This modeling, or variants of it, address aims 3 and 4.

The overall analytic structure requires strategic use of different dummy contrasts and limited information estimation of various MSEM models that map onto the questions dictated by the aims, coupled with judicious use of mediational tests linked to joint significance testing. An alternative analytic structure is to work with all four time periods simultaneously in a classic growth curve model and then to model the linear growth in variables as a function of the provider and clinic variables in strategically defined limited information estimation frameworks dictated by the overall model structure in Fig. 2. We also will explore this approach. Analyses of variable importance (Fig. 1) have been summarized by Tonidandel et al. [58]. Of the two major tools to address the issue (dominance analysis and relative weight analysis), we will use relative weight analysis to gain perspectives because of the less intense computational demands that it makes and its overall flexibility. Missing data will be treated using Full Information Maximum Likelihood (FIML) methods.

A Data Monitoring and Review Board is responsible for reviewing this study’s data to ensure quality and integrity, the safety of participants and study progress, and to make any determinations regarding potential changes to the study. This is comprised of the investigative team with ongoing support from New York University’s Institutional Review Board (IRB). The DMRB meets on a quarterly basis under normal circumstances, or within two business days should an adverse event occur. This process is independent from the sponsors, although they will be notified in the case of a significant change to study protocol or in the case of an adverse event.

### Ethical issues

There are potential but minor risks for providers associated with participating in the study, specifically, feeling coerced into participating, loss of confidentiality, and experiencing discomfort. These risks are addressed in several ways: potential participants are assured that participation is voluntary and will not affect their employment status, all study materials are coded with ID numbers and do not include identifying information, and all data will be maintained in password-protected computer files.

Among caregivers, there is a risk that they may feel uncomfortable with some of the questions in the assessment measures. However, research staff clarify that they do not need to answer any question that makes them feel uncomfortable. The risk to loss of confidentiality is also present for caregivers and is addressed following the same precautions and guidelines used for protecting provider confidentiality. Finally, there is a potential risk that symptoms may worsen as a consequence of the progression of a serious mental health issue or response to a service provided. In the event that this happens, study participation will be halted and appropriate care provided immediately in accordance with clinic procedure.

## Discussion

This study represents an innovative approach to examine the uptake and sustainment of a National Registry of Evidence-based Programs and Practices (NREPP)-approved, evidence-based treatment for child DBDs, and the organizational factors that impede or facilitate adoption of EBPs in public mental health settings. This study will also examine a novel approach to adapt an EBP in a structured way, in order to enhance its fit with the needs of the populations being served and the organizational context. One of the strengths of this study is that it will test uptake across the entire child-serving, public mental health service system in the five boroughs of NYC.

The study aims to generate knowledge needed to address seemingly intractable urban service delivery challenges, including lack of engagement of low-income youth with serious DBDs and their families, too few clinics offering family based, evidence-informed services; lack of scalable, empirically supported interventions designed for resource-strapped child settings; and few empirically supported options for public policy-makers to support the uptake and integration of service innovations within behavioral health care.

### Trial status

**Fig. 3:**
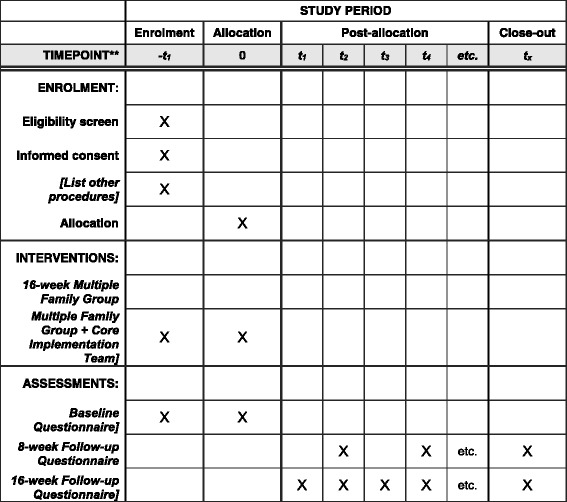
SPIRIT Checklist

Clinic recruitment for standard care has been completed. Data collection has been initiated, and one third (*n* = 792) of the sample of providers and caregivers have been enrolled. Clinic recruitment for MFG and MFG + CIT have been initiated, and some clinics have already completed training and have participated in adaptation meetings. Recruitment of clinics for the experimental arms will be ongoing until enrollment targets are met. Groups for both MFG and MFG + CIT are expected to begin in the first trimester of 2017. A populated Standard Protocol Items: Recommendations for Interventional Trials (SPIRIT) Checklist (see Additional file 1) and Figure ( see Fig. 3) for all study protocols are included as an additional file (see Additional file 1 and Fig. 3). A composition of the data monitoring commitee has also been included (see Additional file 2).

## Additional files

Additional file 1: SPIRIT Checklist. List of where to locate recommended items to address in a clinical trial protocol and related documents. (DOC 122 kb)

Additional file 2: Data Monitoring Committee Information. Information regarding the Data Monitoring Committee for this study. (DOC 15 kb)

## Abbreviations

APQ-9: Alabama Parenting Questionnaire nine-item short form
CESD: Center for Epidemiologic Studies Depression Scale
CIT: Clinic Implementation Team
DBD: Disruptive behavior disorder
EBP: Evidence-based practice
FAD: McMaster Family Assessment Device
IOWA CRS: Iowa Conners Rating Scale
IRS: Impairment Rating Scale
KBT: Kazdin Barriers to Treatment
MFG: Multiple Family Group
MSEM: Multi-level Structural Equation Modeling
MSPSS: Multidimensional Scale of Perceived Social Support
NREPP: National Registry of Evidence-based Programs and Practices
NYC: New York City
NYSOMH: New York State Office of Mental Health
OD: Oppositional defiant
ODD: Oppositional defiant disorder
PSI: Parenting Stress Index
SAMHSA: Substance Abuse and Mental Health Services Administration
SEM: Structural Equation Modeling
TCU ORC: Texas Christian University Organizational Readiness for Change
