# Supplementary material for: Testing the 4Rs and 2Ss Multiple Family Group intervention: study protocol for a randomized controlled trial
Source: Trials. 2017 Dec 4;18:588. doi: 10.1186/s13063-017-2331-7 (PMC5716003; doi:10.1186/s13063-017-2331-7)
Supplement: Supplementary file 2 — Data Monitoring Committee Information. Information regarding the Data Monitoring Committee for this study. (DOC 15 kb) [file 13063_2017_2331_MOESM2_ESM.docx]

**Composition of Data Monitoring Committee**

Consistent with NIMH policy, the [Principal Investigator (PI: McKay)](http://grants.nih.gov/grants/glossary.htm#PrincipalInvestigatorWorkinProgress(PIW)) is responsible for the clinical management of participants and accurate written documentation, investigation, and follow-up of all possible study-related adverse events (AE). The investigator is also responsible for describing and adhering to the procedures for identifying, monitoring, and reporting reportable events, including AEs, Serious Adverse Evens (SAEs), death, Unanticipated Problems, protocol violations, non-compliance, suspensions and terminations. The following process and timeliness for collecting and reporting these evens are as follows:

1. Suspensions or terminations: Within three business days of receipt
2. Deaths related to study participation: No later than five business days of the PI first learning of the death
3. Unexpected SAEs related to study participations: Within 10 business days of the PI becoming aware of the event.
4. Serious or continuing noncompliance: Within 10 business days of IRB determination.
5. Expected AEs and SAEs: Annually, included within the annual progress report.
6. Protocol violations: With the annual progress report.

All reports will be made in writing to the NIMH Program Official (PO). These reports will indicate that the monitoring entities (i.e., the PI and IRB, ISM and/or DSMB) and appropriate regulatory entities (e.g., OHRP, FDA) have been notified in accordance with the approved monitoring plan and federal regulations. Reports will be submitted to the monitoring entity at least annually on a schedule determined by the monitoring entity’s policy. Monitoring entities may require more frequent reporting.

Documentation will include identifying information for the research protocol (e.g., the investigator’s name, project title, the grant/contract number), the data the event occurred and the data the PI became aware of the event, a detailed description of the event and impact on the participants, a detailed description of measures taken, confirmation that the appropriate monitoring entities and regulatory bodies have been monitored, and a description of any changes to the protocol or other corrective actions that have been taken or will be undertaken in response to the event.
